# Supplementary material for: Results and evaluation of the expansion of a model of comprehensive care for Chagas disease within the National Health System: The Bolivian Chagas network
Source: PLoS Negl Trop Dis. 2022 Feb 17;16(2):e0010072. doi: 10.1371/journal.pntd.0010072 (PMC8853485; doi:10.1371/journal.pntd.0010072)
Supplement: S1 Text — (DOCX) [file pntd.0010072.s001.docx]

**S1. Supplementary results: Identification of the actions needed for scaling-up following steps 1 to 8**

We analyzed the implementation of the Chagas Platform strategy and the scaling up process (Chagas Healthcare Network) using the guideline “Nine steps for developing a scaling-up strategy” developed by ExpandNet^1,2^. Here, we describe each of these steps, following the format provided by the guideline (stating attributes and key questions), and also analyzing the innovation and the actions needed.

### Step 1. Planning actions to increase the scalability of the innovation

The innovation refers to health interventions and/or other practices that are being scaled up. We identify components of the innovation by answering the question “What were the activities necessary to put the innovation into place?”.

By retrospectively analyzing the Chagas Platform as the innovation, we found attributes that led to its successful expansion.

The innovation (Chagas Platform) addressed the burden of Chagas disease (CD) in selected areas of Bolivia with a higher prevalence. The comprehensive healthcare protocol for CD was designed based on Bolivian national guidelines and modified in order to make them more efficient locally. For its implementation, no highly sophisticated technical equipment was needed. Interventions were prioritized in agreement with the user organization, resulting in no conflicts during their implementation. Major resources in terms of personnel and equipment were necessary for Chagas Platform implementation. However, during the scaling up, the model was embedded into primary centers of the Bolivian healthcare system without requiring major additional human or financial resources. Adapting protocols and local referral and counter-referral pathways was key during the process.

Chagas Platform results were well documented and proved the efficacy of the vertical strategy, both in rural and urban areas; these results were communicated widely at local and international level.

Although the Chagas Platform approach is more cost-effective than non-intervention^3,4^, cost-effectiveness should be studied in the intervention area.

The Chagas Platform model allowed for progressive adaptation to new contexts, for which working with local stakeholders was key, in order to transfer leadership to them gradually and prioritize intervention areas. In one out of the three rural areas in which it was tested, a horizontal strategy emerged spontaneously responding to local needs.

Although the model showed high efficacy in terms of coverage, there is no a specific tool to measure its impact, and future work could address the development of such a tool.

| **ATTRIBUTE** | **Key questions** | **Chagas Platform as innovation to scale up** | **Chagas Platform model action needed** |
| --- | --- | --- | --- |
| **C - Credibility** | 1. Have results of pilot testing of the innovation been documented?  2. Is further evidence/better documentation needed?  3. Has the innovation been tested in the type of setting where it will be scaled up? | 1. Yes: several concise reports were published on indicators and outcomes of the process and model. They were readily shared with key stakeholders. Documents included Chagas Platform results in terms of coverage of CD healthcare and reports on quality of process.  2. No: there is enough evidence to prove the efficacy of the model as a vertical strategy.  3. Yes: it was tested as a vertical strategy in three different rural areas, and, in one of them, a horizontal strategy emerged responding to local needs. | 1. and 2. Documents should be shared with other stakeholders at national level.  Although the model showed high efficacy in terms of coverage, there is no a specific tool to measure its impact, and future work could address the develop of such tool. |
| **O - Observability** | How observable are results? | Results of the vertical phase were shared with local and international stakeholders, through reports, dissemination material, and ad hoc visits to Chagas Platform centers in all the departments. | No action needed |
| **R - Relevance** | Does the innovation address a felt need, persistent problem or policy priority? | Yes: CD affects 10–20% of the Bolivian population, with prevalence being higher in the intervention areas selected^5^.  Management of CD has been a priority for the Bolivian Ministry of Health (MoH) since March 2006 (law 3374)^6^ | No action needed |
| **R - Relative advantage** | 1. Does the innovation have a relative advantage over existing practices?  2. Is it more cost-effective than existing practices or alternatives? | 1. Yes, and this was shared with all stakeholders. There was an increase in healthcare coverage for CD, mainly for adults.  2. Regarding the intervention area in particular, no cost-effectiveness studies have been carried out. Nevertheless, based on literature on cost-effectiveness^3,4^, due to the high prevalence in the selected area, we speculate that the Chagas Platform approach is more cost-effective than non-intervention. | Cost-effective studies in the innovation area should be carried out. |
| **E - Ease of transfer/ installation** | 1. What degree of change from current norms, practices and level of resources is implied by adopting the innovation?  2. What is the level of technical sophistication needed to introduce the innovation?  3. Does the innovation have the potential for creating conflict in the user organization?  4. Were major additional human or financial resources and commodities needed to introduce the innovation? | 1. The comprehensive healthcare protocol for CD was designed based on Bolivian national guidelines, but practices were modified in order to make them more efficient and effective. In a second phase of the strategy, simplified protocols were adapted, containing the main elements to ensure quality of processes through protocolization.  2. No highly sophisticated technical equipment was needed, which is also lacking in the Bolivian National Health System (NHS).  3. No: Chagas Platform interventions address a current prioritization and problem to be solved by user organizations (e.g., Chagas National Programme [ChNP], Department Chagas Program of Cochabamba)  4. For the vertical Chagas Platform strategy, major resources in terms of personnel and equipment were needed. In the horizontal strategy, no major additional human or financial resources were needed. | The Chagas Platform should be transferred as an innovation, with referral and counter-referral pathways of samples and patients, built together with local healthcare directors and workers. |
| **C - Compatibility** | 1. Is the innovation compatible with current values or services of the user organization?  2. Will it be difficult to maintain the basic values of the innovation as expansion proceeds?  3. Will changes in logistics need to be made to accommodate the innovation?  4. Which components will need local adaptation to be relevant for changes in local context? | 1. Yes: it was a need not covered by the NHS.  2. No: the values were shared with those of the NHS.  3. Yes, a simplification and local adaptation of the model, as well as the generation of referral and counter referral pathways must be implemented.  4.Protocols and local referral and counter-referral pathways. | No action needed |
| **T - Testability** | Can the user organization test the innovation in stages without fully adopting it? | Yes, and this progressive implementation was recommended in order to better adapt the model to real scenarios in each case. | To work together with local stakeholders in order to transfer leadership to them progressively, and prioritize intervention areas. |

Step 2. Increasing the capacity of the user organization to scale-up

Successful scaling-up requires realistic expectations as well as strategies that help to build institutional capacity while expanding an innovation. In our case, the leader user organization was the ChNP, part of the Bolivian MoH. Other public institutions, like universities, were also identified in some context as user organizations, but always under the ChNP leadership.

During Chagas Platform implementation, we identified key personnel interested in the topic at department level and within local universities, and engaged with them in Chagas Platform implementation, and in building the scaling-up approach (Chagas Healthcare Network).

At the beginning of the process, we lacked equipment and/or facilities, and these were reinforced by identifying and prioritizing them together with user organizations. Contributions from user organizations included equipment maintenance, which was more significative during the Chagas Healthcare Network scaling-up.

Regarding personnel training in CD management, we worked together with user organizations to identify areas to be strengthened, and we planned to initially play an active role in training and, afterwards, to serve as advisors. Supervision, monitoring and evaluation should be reinforced in a continuous way in order for the user organizations to be autonomous at the end of the process.

All the interventions proposed were possible without major economic impact on other programs or on current NHS resources.

| **ATTRIBUTE** | **Key questions** | **Chagas Platform as innovation to scale up** | **Chagas Platform model action needed** |
| --- | --- | --- | --- |
| **Perceived need** | 1. Is there a perceived need for the innovation? Does the innovation respond to a policy priority?  2. Are there individuals within the organization who are advocates/champions of the innovation? | 1. The management of CD has been a priority for the MoH since March 2006 (law 3374),^6^ without an operational guideline/plan in place.  2. Yes, we identified key personnel specially interested in the topic at department level and within local universities. We worked together since the establishment of the vertical strategy and through the scaling-up process. | No action needed. |
| **Implementation capacity** | 1. Does the user organization have capacity in: technical skills, training, logistics/supplies, supervision, leadership/coordination, monitoring/evaluation, physical facilities and equipment, values supportive of the innovation, human resources the policy and legal framework necessary to introduce the innovation?  2. Did the pilot project test ways to strengthen the capacity of the user organization?  3. Can the user organization absorb this innovation without negative impact on other programmes and services? | 1. Not all users organizations selected in each area fulfilled all the criteria in terms of capacity at the beginning (pilot project). Several pieces of equipment/or facilities were lacking at the beginning of the process, and through a rational identification and prioritization with user organizations, the project reinforced these areas. Contributions from user organizations included equipment maintenance.  2. Yes: together with user organizations, the project’s coordinators worked on identifying areas to be strengthened, and planned to play first an active role in training and then, to have an advising role.  3. Yes, without major impact on other programs or on the NHS current resources. | 1 and 2. Even if the NHS is able to carry out the strategies in many areas, supervision, monitorization and evaluation should be reinforced continuously. Training of local management personnel at different levels on these capacity areas should be reinforced in order for the user organization to be autonomous at the end of the process. |
| **Timing and circumstances** | 1. Are there impending changes within the user organization that will affect scaling up?  2. Do these changes provide opportunities or constraints? | 1. Yes, there were changes in laws during the scaling-up process. CD affects 10–20% of the Bolivian population, and prevalence of the disease is even higher in intervention areas selected.  2. New laws allowed municipal and department governments to make operational decisions, which facilitated the scaling-up process. | No action needed. |

### Step 3. Assessing the environment and planning actions to increase the potential for scaling-up success

The conditions and institutions external to the user organization (environment) that affect or are affected by the innovation scaling-up may change. Realistic expectations on the magnitude of scaling-up and its success require environmental assessment as an ongoing process so that the scaling-up strategy is adjusted to changing circumstances. The environment includes policy and politics, bureaucracy, the health sector, socioeconomical and cultural context and people’s needs and rights. Anticipating changes in the environment and their potential impact on scaling-up is essential.

The political situation in Bolivia was unstable during the eight-year process of implementation and scaling up, but the coordination team, together with local stakeholders successfully implemented the model despite this barrier. Nevertheless, results will be improved through better coordination with new and synergic initiatives in Bolivia, in order to favor economies of scale. The health sector was the main supporter of Chagas Platform implementation at several levels. However, within some specific departments, mainly at national level, health sector personnel were the barrier to establishing pilot projects and the scaling-up. Better communication between operational and high-level policy makers would have been key in improving their understanding of the Chagas Platform model. During the two phases of the strategy, working together with local authorities resulted in better outcomes and was effective in 1) keeping champions engaged, and 2) neutralizing opponents. During the process, local healthcare authorities from areas that were not initially selected requested to be included in Chagas Healthcare Network.

The demand from civil society for diagnosis and treatment for CD was key, as well as the strategies that increased information among people at risk of having CD. Connections with patient organizations and civil society organizations were helpful, as well as the work promoted with local universities. The strategy could be improved by reinforcing the information, education and communication strategy with messages and materials created in collaboration with civil society.

| **Key questions** | **Chagas Platform as innovation to scale up** | **Chagas Platform model action needed** |
| --- | --- | --- |
| 1. Where in each dimension of the environment is there support or opposition for the innovation?  Which stakeholders need to be engaged?  2. What informal and political connections can be helpful?  3. How can champions be recruited? How can opponents be neutralized or co-opted?  4. Are opportunities or constraints likely to change as scaling up proceeds? Where is there stability and what aspects are most likely to change?  5. Are there related initiatives that could serve to expand the innovation? | 1. The health sector was the main supporter on different levels. However, within some specific departments, mainly at national level, the health sector personnel were the barrier to establish pilot projects and the scaling-up.  The population’s demand for diagnosis and treatment for CD was key, as well as the strategies to increase information among people at risk of having the disease.  Involvement by other Ministries (other than Health) in the development and implementation of the model in both stages was lower than had been projected.  2. Connections with patient organizations and civil society organizations were helpful, as well as the work coordinated and implemented with local universities.  3. During the two phases of the strategy, working together with local authorities resulted in better outcomes and was effective in 1) keeping champions engaged, and 2) neutralizing opponents.  4. The political situation in Bolivian was very unstable during the eight-year process of implementation and scaling-up. Despite this, the coordination team, together with local stakeholders successfully implemented the model.  5. Local healthcare authorities from areas outside those where we carried out the intervention requested to be included. | 1. To promote better communication between operational and high-level policy-makers, in order to improve their understanding of the Chagas Platform model.  To reinforce the information, education and communication strategy with messages and materials created in collaboration with civil society.  To promote intersectoral communication, and engagement with other sectors, such as the Ministry of Education (including Research and Innovation sectors), and the Ministry of Housing.  2. Reinforce work with civil society and promote connections with civil society organizations as a key point for success.  5. Try to better coordinate with new and synergic initiatives in Bolivia in order to favor economies of scale. |

### Step 4. Increasing the capacity of the resource team to support scaling-up.

The resource team refers to individuals and organizations that seek to promote and facilitate wider use of the innovation. In this project, the technical knowledge and leadership was provided by Bolivian and international universities and research institutions. In terms of technical support, specialists from Hospital Clínic in Barcelona (from International Health, Cardiology and Gastroenterology Departments) served as advisors to Bolivian specialists and the implementation team.

The implementation on financial support came, in the vertical phase (Chagas Platform), almost exclusively from Agencia Española de Cooperación Internacional para el Desarrollo (AECID) and, to a lesser extent, other cooperation agencies. During scaling-up (Chagas Healthcare Network), funds came from AECID, the Bolivian NHS, and municipalities in the intervention areas. Additionally, other funders at local and international level like Probitas Foundation, Drugs for Neglected Diseases Initiative (DNDi) and Mundo Sano foundation supported activities, mainly linked with training and research.

During the whole period, a stable, multidisciplinary, strong and highly motivated resource team was built, with the appropriate skills to perform the tasks necessary to scale up the model. Continuous training programs and periodic training events were offered to other stakeholders in order to facilitate scaling-up.

Organization of the resource team was established on two levels: the coordination team and operational team, the latter including several coordination team members. The coordination team was advised by a steering committee that included members from funders, MoH at national and department level, and representatives of local and international universities. Ongoing communication between the different levels facilitated an in-depth understanding of the strengths and weaknesses of the interventions and the possible challenges at the beginning and during scaling-up.

Operational members of the resource team in charge of scaling-up in the second phase worked together with management personnel in the departments. This facilitated communication for the implementation, evaluation and training to support health system monitors. The close informal relationship established at operational level between project coordinators and main user organizations facilitated scaling-up.

It was key to have personnel with previous experience in scaling-up, advocacy and policy development, and a team large enough to provide support in Chagas Platform implementation, and scaling-up (including Bolivian Healthcare personnel).

We identified gender perspective as a weakness in the local resource team; further steps should focus on addressing this in the scaling-up process and in all other contexts.

Although the model was initially created by the resource team in the context of the project, it is the user organization who owns the scaling-up process and not to the resource team. In the case of Chagas Healthcare Network, although the innovation has been successfully incorporated into policy, programs and services in many intervention areas, the process is still ongoing in others, and should be reinforced in order ensure its implementation and sustainability. Also, given the frequency of personnel changes at all levels of government, it is important to note that a resource team with continuity ensures sustainability in the face of turnover of key members of the user organization.

| **ATTRIBUTE** | **Key questions** | **Chagas Platform as innovation to scale up** | **Chagas Platform model action needed** |
| --- | --- | --- | --- |
| **Leadership and credibility** | Are needed skills adequately represented to support: training, relevant clinical service provision, strategic management, advocacy, human rights and gender perspectives, research, monitoring and evaluation, resource mobilization? | The weaknesses identified in the local resource team regarded gender perspective, advocacy and resource mobilization. | Reinforcement of actions to address gender perspective as a transversal approach during scaling-up and in other contexts.  To increase advocacy efforts aimed at stakeholders, including civil society.  To strengthen skills in resource mobilization to ensure sustainability of the scaling-up. |
| **Experience** | Does the team have experience with scaling up, advocacy or policy development? | Yes, part of the team was experienced in this. | No action needed |
| **Size** | Is the team large enough given the amount of support, training, advocacy and networking that will be needed? | Yes, it was large enough to support both phases, and included Bolivian Healthcare personnel | No action needed |
| **Resources** | Are resources available to support the work of the resource team? | Yes, partially | To search for funding at municipal levels (domestic incomes), where resources are available. |
| **Stability** | Are key members of the team likely to continue in this role throughout the scaling-up process? | Yes | No action needed |

### Step 5. Making strategic choices to support vertical scaling-up (institutionalization)

Although the strategy for scaling up our model was mainly horizontal, an institutionalization process of the pilot project was needed before this could occur. Institutionalization requires knowledge related to health systems planning, budgetary cycles, financing, program structures, management, human resources, logistics and information needs. Vertical scaling-up is very important because following Government endorsement of the innovation, there is usually a political commitment to making the necessary policy, legal and health system changes. Rapid expansion with a horizontal strategy without previous institutionalization steps leads, in many cases, to a weak and unstable intervention without local user organizations support.

In our case, the government was interested in the model from its inception, and the vertical scaling-up included adoption and certification of the Chagas Platform model by the Chagas National Programme, with a recommendation for its expansion. At this point, specific actions were needed to incorporate the innovation into the health system at the national, department and municipal level, but under a previous alignment and recognition of positive impact.

To this end, ChNP at national, department and municipal level should be involved in the institutionalization model. Updated guidelines of comprehensive CD management should be published and endorsed by the Bolivian MoH. Regarding facilities, the NHS budget should increase in order to cover diagnosis and treatment costs (following Bolivian ChNP guidelines) in Chagas Platform centers: there is a need for a specialized team to work at each Chagas Platform center to institutionalize the vertical model (a minimum three healthcare workers with different profiles: physician, laboratory technician and nurse). The existing infrastructure belonged to the user organizations and the equipment was provided by the project.

Acquisition and distribution routes of specific drugs (benznidazole and nifurtimox) and reagents should be reviewed, and better procurement plan forecasting demand should be implemented.

In terms of data management, although the Chagas Platform database variables and information system were built taking into account information needed by the NHS, the Chagas Platform information system should be more accurately adapted. Tools and supervision strategies were designed together with Department Chagas Programmes, but their approval as national/department practices should be carried out. Staff evaluation is currently undertaken at the end of training in Platform centers, and certified by Chagas Department Programme. A periodic evaluation of staff is recommended.

For further improvement, more comprehensive Information, Education and Communication (IEC) material should be created together with target populations and endorsed by NHS institutions.

| **Category of change** | **Change needed**  **(yes/no/unknown)** | **Chagas Platform model specific changes needed or how need should be assessed** |
| --- | --- | --- |
| **Policy** | No | - |
| **Political commitment** | Yes | All levels and departments of ChNP at national, department and municipal level should be involved in the model of institutionalization. |
| **Legal change** | No | - |
| **Regulations, norms and guidelines** | Yes | There are no approved guidelines of CD management, although the former ones were reviewed and completed by the Chagas Platform coordination team together with ChNP direction. |
| **Financing and budgets** | Yes | Based on population demand and the burden of disease in Bolivia, the NHS budget should increase in order to cover diagnosis and treatment costs (following Bolivian ChNP guidelines).  To institutionalize the model, part of the budget should be allocated to a specialized team to work on the Platform (minimum of three healthcare workers). The existing infrastructure belonged to user organizations and equipment was provided by the project. |
| **Logistics** | Yes | In terms of healthcare coverage for CD, specific drugs and reagent acquisition and distribution routes should be reviewed. Forecasting demand and the procurement plan should be improved. |
| **Management information systems** | Yes | The Chagas Platform information system should be adapted to that of the NHS. Platform database variables and information system were built taking into account information needed by the NHS but this could be further improved. |
| **Supervision** | Yes | Tools and supervision strategies were designed together with Department Chagas Programmes, but their approval as national/department practices should be carried out. |
| **Staff evaluation, performance incentives** | Yes | Staff is currently evaluated at the end of the training in Platform centers, and certified by Chagas Department Programme. A periodic evaluation of staff is recommended. |
| **Training curricula and approaches** | Yes | There should be continuous training in CD and public health, also to be included in health degrees. |
| **Health workforce changes** | Yes | The workforce should be changed regarding misconceptions about CD. |
| **IEC materials** | Yes | More comprehensive IEC material should be created together with the target populations. |

### Step 6. Making strategic choices to support horizontal scaling-up (expansion/replication): The Chagas Healthcare Network

Innovations may be replicated in different locations and can be extended to serve large, diverse population groups. Applicability and scalability were core elements of the strategy selected to scale up the Chagas Platform. The expansion process required exhaustive adaptation of the model to the different environmental contexts that were selected together with the Chagas Department Programme: municipalities of the Cochabamba, Tarija and Chuquisaca departments.

Key elements to achieve Chagas Platform scaling-up and to make up the Chagas Healthcare network were analyzed in terms of 1) dissemination and advocacy, 2) organizational process, 3) resource mobilization, and 4) monitoring and evaluation.

Regarding Dissemination and advocacy, publication of the vertical strategy (Chagas Platform) as well as other dissemination materials were very useful to illustrate the model.^7^ For better dissemination, we suggest developing additional materials in different formats adapted to different audiences.

Additionally, IEC material was created together with user organizations and transferred to them, although messages should be adapted to different audiences in a continuous exercise.

Healthcare workers received theoretical training and a practical stage before starting the scaling up in the Chagas Platform reference centers established in the first (vertical) stage of implementation. Training was certified by Chagas Department Programme and was offered to personnel designated by them. When implemented, technical assistance was based on expert advice in the Chagas Healthcare network, with the support of Chagas Platform personnel.

During the Chagas Platform scaling-up, where possible, a participatory approach was taken involving all levels of the health system and including members of the community, which contributed to community empowerment and fostered ownership of the innovation.

Regarding the organizational process, the Chagas Platform expansion to the Chagas Healthcare Network took four years, once the Chagas Platform model was accepted, and the process is still ongoing: different areas of intervention are in different stages of maturity in terms of their adoption of the model. The progressive scaling-up of the Chagas Platform is the short and mid-term expectation; the long-term goal is to continue expanding the model via the NHS without external support, consolidating growth of the Chagas Healthcare Network.

Participation with other stakeholders in critical evaluation and deciding new directions of the model is highly recommended, as well reinforcing the work together with new partners in order to better support scaling-up.

The results obtained from preliminary monitoring and evaluation were used to adjust the scaling-up strategy. A key aspect for the success of the expansion was starting the process in areas with strong organizational capacity and leadership (for example, Punata area). A gradual expansion was carried out with the pace of implementation tailored to each geographical area and adapted to their specific characteristics. In our experience, this approach facilitates sustainability in terms of better alignment with politics, health policy, and available local resources in each area.

Additionally, the process and the model should undergo periodic external evaluation.

During the initial phase of the vertical scale-up of the Chagas Platform model, AECID and other external funders were the main source of financial support. However, in the expansion phase, costs were gradually allocated to national and local budgets.

| **Strategic**  **choice area** | **Key questions** | **Chagas Platform in the process of scaling-up** | **Chagas Platform model action needed to improve scaling-up** |
| --- | --- | --- | --- |
| **Dissemination and advocacy** | 1. What political, personal, or other informal channels and relationships can be used to convince new areas (districts, municipalities etc.) to  introduce the innovation?  2. How will the innovation be  communicated/transferred (training, technical assistance, peer to peer  approaches, Information, Education and Communication (IEC) materials, mass  media, reports, policy briefs)?  3. Is communication about key aspects of the innovation appropriately tailored and presented to different audiences and conveyed in clear and concise messages? | 1. In our case, department directors of Chagas Programmes were key actors, as well as health directors at municipalities and municipal hospital management.  2. In terms of transference, a system of training the trainers was implemented. Technical assistance was based on expert advice in the Chagas Healthcare network, with the support of Chagas Platform personnel. IEC material was created with and transferred to the user organizations. A final report was designed and distributed.  3. Yes, but messages should be better adapted to different audiences. | 1. IEC strategies should be reinforced to ensure alignment with communities and their leaders on actions taken to increase access to CD comprehensive care.  2. To continue giving advice to Chagas Healthcare Network.  To improve current communication plan and increase actions to make it more robust and aligned with stakeholders’ priorities and communication channels.  3. To better communicate processes and results to different audiences and in different formats adapted to them. |
| **Organizational process** | 1. How many sites are expected to adopt the innovation?  2. What is the time period during which expansion will take place? Will implementation be phased or rapid?  3. What are feasible short- term expectations, what are mid-term expectations and what are long-term goals?  4. Are there major differences among sites? If so, what adaptations to the innovation are needed?  5. Will new partners be brought in to support or implement scaling up?  6. Will the approach be participatory? | 1. The specific information on this topic is in the results section. Expansion areas were defined together with user organizations, and are currently more than expected due to spontaneous scaling up (see step 8)  2. Platform expansion to the Bolivian Primary Healthcare centers started in 2014 in a staged manner. Different areas of intervention are in different stages of maturity in terms of their adoption of the model.  3. Short and mid-term expectations were to expand the model in different settings and to prove that adaptation of the model was possible, with a positive impact. The long-term goal is to continue expanding the model via the NHS without external support, and to consolidate the expansion of the Chagas Healthcare Network.  4. Yes, but the model has been adapted to each site with the Platform’s twelve steps (Figure 2, main text)  5. Several new partners (mainly municipalities of areas not initially included) have been identified to support Platform scaling up, with the aim that they join the Chagas network. Local Universities who participated in a minor way in the first phase are also interested in support the scaling up process, as is the Planification Department at the national level.  6. Up until now, all stakeholders were involved in the process. | 2. Close follow-up of areas in which the model has been recently expanded, and monitor new areas proposed.  3. To give advice to user organizations to support the Platform model consolidation in the Chagas Network.  4. To take in account differences in future areas and apply the Platform’s twelve steps in order to ensure adaptation.  5.To reinforce the work together with new partners in order to better support scaling-up  6. To promote participation with other stakeholders in critical evaluation and deciding new directions of the model, if needed. |
| **Costs/ resource mobilization** | 1. Will the costs of expansion be the same for each new area?  2. Are economies of scale possible?  3. Can expansion be more efficiently organized?  4. Are resources for expansion available or do they need to be mobilized? | 1. The cost in each area was slightly different.  2. Yes. Before scaling up the model, a business plan and budget were developed in conjunction with local authorities. The proposed expenditure was included in local authorities’ annual budgets (with a signed commitment to participate for at least five years)  3. A specific analysis in terms of efficiency was made in each area, taking advantage of referral and counter-referral pathways.  4. The resources for expansion at municipal levels are available and already committed to give sustainability to Chagas Network. | 3. Efficiency should be continuously evaluated in each area, including cost-benefit studies.  4. Resources at national level (ChNP) to cover diagnosis reagents and treatment expenses should be increased. |
| **Monitoring and evaluation** | 1.How will the process, outcomes and impact of moving to scale be monitored and evaluated?  2. Are the innovations continuing to have the intended outcomes and impacts that were demonstrated in the pilot? | 1. Monitoring and evaluation were carried out in terms of screening, diagnosis and treatment coverage, by gender and age, in each specific area. Data were supervised and managed by Department and National Programmes. Simplified databases were designed for this purpose in the context of the project.  2. Yes | 1. In order to better assess impact,  qualitative studies should be included to gain insight into the process and barriers of expansion. The process and the model should periodically evaluated. |

### Step 7. Determining the role of diversification

Diversification or functional scaling-up refers to testing additional innovations to be scaled up besides the main one. During the scaling-up process, new components and a new tool were added to actions towards the same goal (to improve comprehensive healthcare for CD in Bolivia).

The additional innovations were:

a) Extension of activities to the pediatric population. This was requested by user organizations and, due to the benefits of early diagnosis and treatment, especially in children younger than one year of age, the impact was positive and helped to consolidate the model.

b) Decentralization of diagnostic processes from the specialized diagnosis centers to primary healthcare centers by reinforcing diagnosis facilities. This took place through a technical collaboration with PROBITAS Foundation (which also funded the project). Additionally, the improvement of diagnosis facilities also allows for better diagnosis of other prevalent and neglected diseases. A laboratory software in the context of PROBITAS Global Laboratory Initiative was created with this purpose, and laboratory personnel were specifically trained in the use of the new techniques and the software.^8^

c) Development of Xtrategy^9^, a new tool in the context of an ancillary project obtained to reinforce the implementation of the Chagas Healthcare Network. Xtrategy is a software that supports healthcare decisions by modeling the complexity of the health system for efficient Public Health planning. The tool offers insights on potential impact of actions on control of CD in concrete scenarios. Xtrategy could be used at different stages of decision makers, from municipal to national level, in order to coordinate actions to address health issues. It was designed and tested to support CD control. After being piloted in Chagas Healthcare Network selected sites and in non-endemic countries, its use has been expanded to other epidemiological contexts in Bolivia with different needs in terms of control of CD through a pilot project. The tool could be adapted to other neglected and/or prevalent diseases affecting a specific geographical area.

### Step 8. Planning actions to address spontaneous scaling-up

Spontaneous scaling-up refers to diffusion of the innovation without deliberate guidance. In our case, as showed in the results section of the manuscript, several municipal areas and primary care centers not selected initially asked to be included in the network. All centers that requested it, were included in the network under the premises that: a) the Chagas Platform model would be adapted under Chagas Department Programme supervision, supported by project personnel; and b) that there was adequate financial support for the new actions to be implemented to ensure sustainability.

The results and impact of spontaneous scaling-up were analyzed in two ways. The results were analyzed as part of the total intervention and also separately, in order to assess if all the elements of Platform model were replicated and if results in these cases were similar to those of centers included in the original planned intervention.

All components related to healthcare were scaled up, except for research and, in some cases, laboratory facilities. A key point is the need for initial advising in monitoring and evaluation in order to ensure quality of the process; these ideally would decrease in favor of municipal and department sustainability and autonomy.

| **Strategic**  **choice area** | **Key questions to be asked if there is**  **evidence that spontaneous scaling**  **up is taking place** | **Chagas Platform in the process of scaling-up** | **From Chagas Platform, it would be desirable find ways to…** |
| --- | --- | --- | --- |
| **Dissemination** | 1. How is the innovation spontaneously disseminated?  2. Are all the components of the innovation being scaled-up or only some aspects of it?  3 Can spontaneous diffusion of the innovation potentially replace the process of guided scaling up? | 1. Via the information on efficiency of the model shared in “Mesas de Salud” and other meetings in which municipalities, healthcare workers, and department directors analyzed CD screening, diagnosis and treatment coverage.  2. All components related to healthcare were scaled, except for research and, in some cases, laboratory facilities.  3. Yes, in the future, but the continuous supervision/evaluation of user organizations is currently recommended. | 2, 3. Share and distribute resource materials to facilitate spontaneous scaling-up.  Give advice on development of new IEC materials with civil society and other stakeholders. |
| **Organizational process** | What is the pace and scope of spontaneous scaling up, and how are the human resource and other managerial issues of expansion addressed? | The pace of scaling-up is adapted based on the specific setting. | Give advice to Chagas Department Programme in evaluating requests through the twelve steps process. |
| **Costs/ resource mobilization** | How are the costs of scaling up absorbed when the innovation is spontaneously scaled-up? | Costs must be absorbed by the NHS, mainly by municipalities, according to Bolivian resource distribution. | - |
| **Monitoring and evaluation** | 1. Should spontaneous scaling up be further evaluated?  2. Who should be responsible for monitoring and evaluation? | 1. Yes, because new centers are going to be included in the future in the Chagas Healthcare Network.  2. Chagas Department Programme. | 1 and 2. Continue as advisors of training supervisions. |

# **REFERENCES**

1. Simmons R, Fajans P, Ghiron L. Introduction. In: Simmons R, Fajans P, Ghiron L, eds. Scaling up health service delivery: from pilot innovations to policies and programmes. Geneva, World Health Organization 2007;vii–xvii.
2. Nine steps for developing a scaling-up strategy. Department of Reproductive Health and Research. WHO Library Cataloguing-in-Publication Data.2010
3. Requena-Méndez A, Bussion S, Aldasoro E, et al. [Cost-effectiveness of Chagas disease screening in Latin American migrants at primary health-care centres in Europe: a Markov model analysis.](https://www.ncbi.nlm.nih.gov/pubmed/28256340) Lancet Glob Health. 2017 Apr;5(4):e439-e447. doi: 10.1016/S2214-109X(17)30073-6.
4. Bartsch SM, Avelis CM, Asti L,et al. [The economic value of identifying and treating Chagas disease patients earlier and the impact on Trypanosoma cruzi transmission.](https://www.ncbi.nlm.nih.gov/pubmed/30395603) PLoS Negl Trop Dis. 2018 Nov 5;12(11):e0006809. doi: 10.1371/journal.pntd.0006809.
5. World Health Organization. Chagas diseases in Latin America: an epidemiological update based on 2010 estimates. Weekly Epidemiological Record No6 2015; 90:33-44.
6. Ramírez-Valverde S, Novillo-Aguilar E, Alberto Díaz R, et al. Ley No 3374 Ley de 23 de Marzo de 2006. In: Bolivia EPd, editor.; 2006.
7. Pinazo MJ, Pinto J, Ortiz L, et al. [A strategy for scaling up access to comprehensive care in adults with Chagas disease in endemic countries: The Bolivian Chagas Platform.](https://www.ncbi.nlm.nih.gov/pubmed/28820896) PLoS Negl Trop Dis 2017;11(8):e0005770.
8. <https://www.fundacionprobitas.org/en/what-is-gli-model> (accessed on 2nd June 2021)
9. <http://xtrategy.coalicionchagas.org/> (accessed on 2^nd^ June 2021).
