# Supplementary material for: Results and evaluation of the expansion of a model of comprehensive care for Chagas disease within the National Health System: The Bolivian Chagas network
Source: PLoS Negl Trop Dis. 2022 Feb 17;16(2):e0010072. doi: 10.1371/journal.pntd.0010072 (PMC8853485; doi:10.1371/journal.pntd.0010072)
Supplement: S1 Table — Finalizing the scaling-up strategy and identifying next steps: Recommended actions in the scaling-up steps. (DOCX) [file pntd.0010072.s002.docx]

**S1 Table. Step 9. Finalizing the scaling-up strategy and identifying next steps: Recommended actions in the scaling-up steps**

We have learnt that an appropriate balance among the elements of the scaling-up system is essential. We have summarized recommended actions in each step (1 to 8) and their degree of priority, to serve as a guide for future work to consolidate the model (Table S1).

| **Broad category** | **Recommendations** | **Degree of priority** |
| --- | --- | --- |
| Step 1. Evaluating the innovation (Planning actions to increase the scalability of the innovation) | Increase activities to share pilot results and documents with other stakeholders at national level. | Moderate |
|  | Carry out cost-effective studies in the innovation area. | Moderate |
|  | Develop, together with local stakeholders, an impact evaluation tool, easy to use and implement. | High |
| Step 2. Increasing the capacity of the user organization to implement scaling up | Continuously reinforce supervision, monitoring and evaluation. Training of local directors at different levels in the field should be a priority in order to encourage user organization autonomy as the end result. | High |
| Step 3. Assessing the environment and planning actions to increase the potential for scaling-up success | Promote better communication between operational/clinical staff and political decision-makers, in order to improve understanding of the Chagas Platform model. | High |
|  | Reinforce the information, education and communication strategy with messages and materials created together with civil society. | High |
|  | Promote intersectoral communication and engagement with other Ministries involved in CD control activities. | Moderate |
|  | Improve coordination with new and synergic initiatives in Bolivia, in order to favor economies of scale. | Moderate |
| Step 4. Increasing the capacity of the resource team to support scaling up | Encourage and include gender perspectives as a transversal approach. | High |
|  | Reinforce advocacy programmes with all stakeholders, including civil society. | High |
|  | Search for funding sources to ensure future sustainability. | Moderate |
| Step 5. Making strategic choices to support vertical scaling up (institutionalization) | Publish guidelines on CD management, relevant to different healthcare levels, and validated by ChNP. | Moderate |
|  | Increase NHS budget to cover diagnosis and treatment costs (following Bolivian ChNP guidelines). (applicable also for step 6) | High |
|  | Adaptation of Platform information system to that of the NHS. | Moderate |
|  | Continuous training in CD and public health and its inclusion in Health degrees (also for step 6). | High |
| Step 6. Making strategic choices to support horizontal scaling up (expansion/replication) | Provide technical advice to ensure quality and sustainability of the Chagas Network. | High |
|  | Promote participation of other stakeholders in critical evaluation and new directions of the model, if needed. | High |
|  | Implement qualitative studies in order to assess impact, to gain insight into the processes of, and barriers to expansion. | Moderate |
| Step 8. Planning actions to address spontaneous scaling up | Share and distribute resource materials to facilitate spontaneous scaling-up. | Moderate |
|  | Give advice on co-creation of new IEC materials with civil society and other stakeholders. | Moderate |
