# Supplementary material for: Results and evaluation of the expansion of a model of comprehensive care for Chagas disease within the National Health System: The Bolivian Chagas network
Source: PLoS Negl Trop Dis. 2022 Feb 17;16(2):e0010072. doi: 10.1371/journal.pntd.0010072 (PMC8853485; doi:10.1371/journal.pntd.0010072)
Supplement: S2 Table — (DOCX) [file pntd.0010072.s003.docx]

## **S2 Table. Antiparasitic treatment and ADRs in Chagas Platforms centers and Chagas network**

|  |  | **Antiparasitic treatment administered**  **(n)** | **Antiparasitic treatment completed**  **(n)** | **ADRs reported**  **(n)** | | | **Cause of treatment discontinuation**  **(n)** | | | **Treatment abandoned by patient**  **(n)** |
| --- | --- | --- | --- | --- | --- | --- | --- | --- | --- | --- |
|  |  |  |  | **Mild** | **Moderate** | **Severe** | **ADRs** | **Pregnancy** | **Other** |  |
| **Chagas Healthcare Network** | F | 1114 | 820 | 217 | 73 | 27 | 157 | 2 | 9 | 126 |
|  | M | 806 | 550 | 123 | 38 | 20 | 89 | - | 8 | 159 |
| **Chagas Platform centers** | F | 6,163 | 5,034 | 2,639 | 898 | 181 | 604 | 25 | 47 | 452 |
|  | M | 3,928 | 3,323 | 1049 | 281 | 125 | 269 | - | 37 | 300 |
| **Total (n, %)** |  | **12,011** | **9,727 (80.9%)** | **4,028 (33.5%)*** | **1,290**  **(10.7%)*** | **353**  **(2.9%)*** | **1,119**  **(9.3%)*** | **27**  **(0.2%)*** | **101**  **(0.8%)*** | **1,037**  **(8.6%)*** |

Abbreviations: ADRs, adverse drug reactions; F, female; M, male; *% of number that initiated treatment
