# Supplementary material for: Results and evaluation of the expansion of a model of comprehensive care for Chagas disease within the National Health System: The Bolivian Chagas network
Source: PLoS Negl Trop Dis. 2022 Feb 17;16(2):e0010072. doi: 10.1371/journal.pntd.0010072 (PMC8853485; doi:10.1371/journal.pntd.0010072)
Supplement: Attachment — Submitted filename: Response to Reviewers.doc [file pntd.0010072.s004.doc]

To the Editors of “**PLoS NTD"**

Barcelona, 10th November 2021

Dear Dr. Natalie Bowman and Dr. Joseph Vinetz, Editors PLoS NTD,

We have read with interest the comments of the reviewers to the manuscript entitled “**Results and evaluation of the expansion of a model of comprehensive care for Chagas disease within the National Health System: the Bolivian Chagas network" (PNTD-D-21-01236) - [EMID:78209386c6be6929]**

As suggested by the reviewers, we have reviewed and modified the version 1.0 of the manuscript. The new version contains the following modifications (line number refers to track-changes version).

Response to reviewer’s comments:

**Reviewer #1 comments and responses**
 Line 184 – in to - correct to into

**R: we have made the correction indicated by R#1 (page 10, line 183)**

 Line 247 Table 3 –  please explain what are the categories “Chagas network” and “Chagas Platform centers” . The title proposes “Chagas network” and in Table 3 there is a division between “Chagas network” and “Chagas Platform”. Please define better these categories.

**R: thank you very much for the comment. The definition of “Chagas Platform centers” and “Chagas (Healthcare) Network” was included in several manuscript sections. The first (Chagas Platform centers), refers to the seven centers linked to the Chagas Platform (specialized reference centers developed in the first phase); and “ Chagas Network” refers to the primary healthcare network implemented during the scaling-up phase of the study. To minimize confusion, we have renamed “Chagas network” category into “Chagas Healthcare Network”, in order to harmonize nomenclature (page 13, line 247 and Table 3 category)**

**Abstract, lines 65-67 and**

**[…]The first stage of the project was to implement a vertical pilot program in order to introduce and consolidate a consensual protocol-based healthcare, working in seven centers (Chagas Platform Centers[…]**

**Author Summary, lines 83-88: […] A two-phase strategy was designed to introduce protocol-based healthcare in Bolivia through prevention, case-management, healthcare professionals training, and community activities. From an initial seven centers in the vertical phase (Chagas Platform centers), 52 healthcare primary healthcare centers adopted CD protocolized care in a second phase (Chagas Healthcare Network) through decentralized, horizontal scaling-up. […]**

**Introduction, lines 113-119: […] With the aim of reinforcing the Chagas National Programme (ChNP) of the Bolivian MoH and improving CD control, we developed the Bolivian Chagas Platform in 2009. [8] The first stage of the project (2009–2014) implemented a vertical pilot program to introduce protocol-based healthcare using a top–down strategy in coordination with local health authorities. [8] The second phase (2015–2018) aimed at scaling up this vertical model to primary healthcare centers with a comprehensive and realistic horizontal approach, simplifying processes, and building the Chagas Healthcare Network (Fig 1). […]**

 Line 295 – please correct  Depatmental Network to Department Network

**R: we have made the correction indicated by R#1 (page 15, line 297)**

 Line 311 – what is ChNP ? Chagas network platform?

**R: we would like to thank R#1 for the comment. ChNP means Chagas National Programme, and in this version we have included the abbreviation at the first time it appears in the main text (page 6, line 113)**

 Line 312 – Please explain this sentence “Although dialogue with local authorities was complicated when addressing intersectoral issues, it led to more robust results when addressing CD.”

**R: in order to give more clarity to the idea expressed in the sentence, we have rephrased it (page 17, lines 333-334)**

**Reviewer #2 comments and responses**

The manuscript titled "Results and evaluation of the expansion of a model of comprehensive care for Chagas disease within the National Health System: the Bolivian Chagas network" is a great description and evaluation about an impressive health primary care model built to achieved health access for people affected by Chagas disease.  I think that for the main purpose of this manuscript do not need any correction, but after read it I have some question of personal interested if authors are willing to answer or discussed:
1)      besides serologies and treatment, do you have any data about EKG or cardiologic clinical evaluation?  Any support/ contacted to transfer patients whose need second or third level health assistance?

**R: we would like thank R#2 for his/her positive evaluation of the manuscript. In regards to the cardiological evaluation, the guideline of patient’s management used in Chagas Platform and adapted to the Chagas Network, included a whole cardiological and digestive evaluation of each patient with confirmed *T. cruzi* infection before starting the treatment, following international guidelines and recommendations. On the other hand, referral and counter-referral flows were established together with local stakeholders (from municipal level to highest complexity centers) in order to manage patients with organ involvement that required specialized healthcare attention as well as for the management of moderate and severe adverse events due to the use of Benznidazole and Nifurtimox. Is has been described in Supplementary material (S1: Steps 1 and 6)**

2)      Paragraph (lines 301-304) “ In terms of healthcare coverage, it is important to highlight that, during the four years of scaling-up, at-risk people managed in the intervention areas increased by 160% with the comprehensive approach proposed by the Chagas Platform, compared to the rates achieved in the eight years of vertical approach”
I understand that much much more people of risk were tested but in table 3, the prevalence of seropositives in the initial Platform were 66,7% while in the scale up network was 18,1 % , both prevalence are really high, but the first one extremely high ( at least one of two people tested were positive!)  Why do you think is this difference prevalence between the platform and the network?

**R: thank you for highlight and note this fact. It has been, indeed a matter of discussion with healthcare authorities in Bolivia. Chagas Platform approach (the vertical one) is more effective, but in terms of sustainability and access is less efficient, given that Chagas Platform centers are only seven in the country, in specific places, depending mainly on external funding and specialized in Chagas Disease management. Thus, data coming from Chagas Platform patient’s prevalence is under a selection bias. People attending specialized centers are in most cases derived from other healthcare centers to be treated, or are relatives of previously diagnosed people, or even people that knew about their diagnosis on Chagas previously and aimed to be treated in the centers. On the contrary, people attending primary healthcare centers belonging to Chagas Healthcare Network are people that usually attend the healthcare center for any reason, and *T.cruzi* screening is included in their regular management, but in a protocolized way. In this case, prevalence observed is closer to reality than the one observed in the Chagas Platform centers.**

**We have included this explanation in the discussion (page 16 and 17, lines 306-325), in order to enrich this section.**

3)      In the same table 3 there is a high difference in people treated between platform (48,6%) and platform ( 8,1%), could be this associated to clinical trials?

**R: Thank you again for the interesting comment. We have included the following information into the explanation mentioned in the previous comment (page 16 and 17, lines 306-325): In terms of access to treatment, absolute number of patients treated in these seven centers is lower than in the network, and it doesn’t mean a problem in terms of drugs stock, and on the other hand, the most of the people attending Chagas Platform centers were young people without treatment contraindications. In the case of Chagas Healthcare Network, regarding specific treatment, the profile of patients attending primary healthcare centers was slightly different from those attending the Chagas Platform, having a percentage of them contraindications to start etiologic treatment due to age and/ or organ involvement.**

4)      Table 4,  1000 people abandoned treatment, may be many reasons but could be possible to know why?  So if there is any cultural o social barrier the health care system could do a better approach.

**R: it is a very interesting point, and even if the specific cause of each patient to stop the treatment by their own decision is registered in clinical record, it wasn’t be analyzed formally. It is a very good topic to include in future research approaches.**

5)      Paragraph (lines 267-271) “In terms of prevention, scaling-up of the entomological surveillance with community engagement achieved higher rates of house infestation reports than with house-by-house vector control by technicians, leading to more effective and sustainable spraying (Rojas Cortez M, et al..)”. It is possible to know if have find infected houses? If yes, when (which year) was the last one find

**R: Yes, people find triatomines, infected and not infected continuously (to the present). There is a recent paper on that topic in which the results of community-based entomological surveillance is showed:**

**Rojas-Cortez M, Pinazo MJ, Gascon J, Gamarra E, Grageda RM, Fernandez R, Rueda E, Pinto J, Anzoleaga HM, Caballero YE, Urioste GS, Sanchez J, Saravia R, Castellon M, Garcia W, Daza LO, Mur IG, Lozano D, Carvalho-Costa FA, Monteiro FA, Torrico F.** [**Community-based entomological surveillance in three Chagas disease-endemic regions in sub-Andean Bolivia.**](https://pubmed.ncbi.nlm.nih.gov/34580735/) **Trans R Soc Trop Med Hyg. 2021 Nov 1;115(11):1251-1259. doi: 10.1093/trstmh/trab150.**

We would like to take the opportunity to thank the reviewers for helping us improve the quality of the manuscript.

Looking forward to your response, we remain at your service for any questions or explanations.

Yours sincerely,

Maria-Jesus Pinazo and co-authors.

ISGlobal, Hospital Clínic-Universitat de Barcelona, Carrer Roselló 132, 08036 Barcelona, Spain.
